# 1661. Incorporating Infection Prevention and Nursing into Antimicrobial Stewardship Handshake Rounds

**DOI:** 10.1093/ofid/ofad500.1494

**Published:** 2023-11-27

**Authors:** Kathleen Martinez, Matthew J Weber, Ann-Christine Nyquist, Sarah K Parker, Sonja I Ziniel

**Affiliations:** Children's Hospital Colorado, Arvada, Colorado; University of Colorado/Children's Hospital Colorado, Aurora, Colorado; University of Colorado/Children's Hospital Colorado, Aurora, Colorado; University of Colorado/Children's Hospital Colorado, Aurora, Colorado; University of Colorado School of Medicine/Children's Hospital Colorado, Aurora, Colorado

## Abstract

**Background:**

Antimicrobial Stewardship Programs’ (ASP) “handshake stewardship” rounds are used in many hospitals to review patient antibiotic prescribing and use in real time. Historically, handshake rounds at our organization have consisted of Infectious Disease providers and pharmacists but have not included infection prevention (IP) and nursing. While recent literature suggests the value of including infection prevention and nurses in ASPs, information is lacking on practical implementation and assessment of such inclusion. The objective of this study was to understand the perceived value of adding IP and nursing to existing “handshake stewardship” rounds lay the groundwork for sustained implementation to improve both antimicrobial stewardship and infection prevention practices hospital wide.

**Methods:**

Surveys to assess provider and nursing perceptions were developed with infection control, nursing, stewardship, and survey specialist input. Data was collected in REDcap from 1,030 physicians and advanced practice providers (APP) and 170 nurses on units where “handshake stewardship” rounds occur daily.

**Results:**

276 respondents completed the 12-question survey (response rate: 23%). 52% of physicians and nurses rated the potential inclusion of an IP as “very useful” or “pretty useful”. 52% of physicians and 48% of nurses responded that including nurses in ASP rounds would be “very useful” or “pretty useful”. Free responses were analyzed for themes.

**Table 1:**
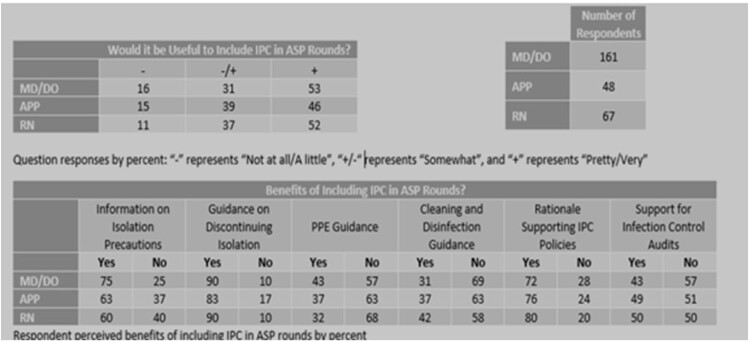
Survey Results

Survey Question: Would it be useful to include Infection Prevention and Control an ASP Rounds?

**Table 2:**
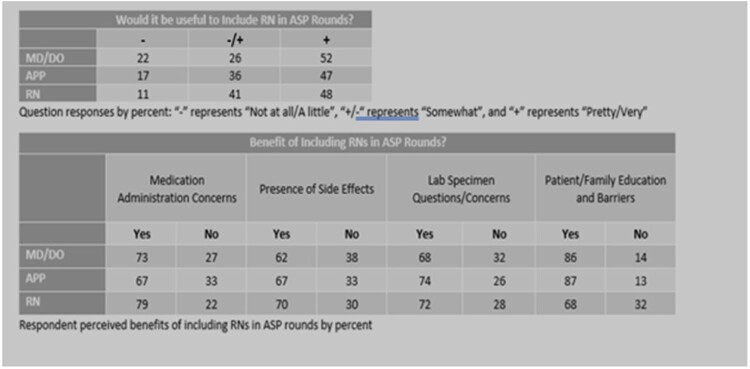
Survey Results

Survey Question: Would it be useful to include RNs in ASP rounds?

**Conclusion:**

Our results demonstrate a mismatch between literature recommendations and survey participants’ perceptions. Despite mixed perceptions of value, nurses’ free text comments reflect interest in learning more about this nursing role. While the usefulness of including IPs was viewed positively in only over 50% of respondents, opportunities for improvement were reflected in the high number of nurses and providers who lacked confidence in knowing IP policies. Results suggest the opportunity for education on the potential contributions of nursing in antimicrobial stewardship and infection prevention, facilitated by IP presence on Handshake Stewardship rounds. The ASP rounding tool can be harnessed to ensure successful integration of nurse and IP specific processes. Tools to assess the success of this initiative are in progress.

**Disclosures:**

**All Authors**: No reported disclosures

